# The Ca^2+^:H^+^ coupling ratio of the plasma membrane calcium ATPase in neurones is little sensitive to changes in external or internal pH

**DOI:** 10.1016/j.ceca.2011.03.004

**Published:** 2011-06

**Authors:** Roger C. Thomas

**Affiliations:** Physiological Laboratory, Department of Physiology, Development and Neuroscience, University of Cambridge, Downing Site, Cambridge CB2 3EG, UK

**Keywords:** Intracellular pH, Neurone modulation, Calcium pump, Extracellular pH, PMCA

## Abstract

To explore the effects of both external and internal pH (pH_o_ and pH_i_) on the coupling between Ca^2+^ extrusion and H^+^ uptake by the PMCA activity in snail neurones H^+^ uptake was assessed by measuring surface pH changes (ΔpH_s_) with pH-sensitive microelectrodes while Ba^2+^ or Ca^2+^ loads were extruded. Ru360 or ruthenium red injection showed that injected Ca^2+^ was partly taken up by mitochondria, but Ca^2+^ entering through channels was not. External pH was changed using a mixture of three buffers to minimise changes in buffering power. With depolarisation-induced Ca^2+^ or Ba^2+^ loads the ΔpH_s_ were not changed significantly over the pH range 6.5–8.5. With Ca^2+^ injections into cells with mitochondrial uptake blocked the ΔpH_s_ were significantly smaller at pH 8.5 than at 7.5, but this could be explained in part by the slower rate of activity of the PMCA. Low intracellular pH also changed the ΔpH_s_ responses to Ca^2+^ injection, but not significantly. Again this may have been due to reduced pump activity at low pH_i_. I conclude that in snail neurones the PMCA coupling ratio is either insensitive or much less sensitive to pH than in red blood cells or barnacle muscle.

## 1 Introduction

Cell calcium must be kept very low to allow fast and efficient intracellular Ca^2+^ signalling. In nerve cells two ATPase pumps are involved in removing Ca^2+^ from the cytoplasm. These are the sarco-endoplasmic reticulum calcium ATPase (SERCA) which drives uptake into the endoplasmic reticulum and the plasma-membrane calcium ATPase (PMCA) which extrudes Ca^2+^ across the plasma membrane. As first shown by Niggli et al. many years ago the latter couples the efflux of Ca^2+^ to the uptake of H^+^ ions [1]. The properties of the PMCA have been reviewed recently [2,3]. The PMCA is inhibited by extracellular alkalinisation and intracellular acidification [4–8]. In some cells there is a second plasma membrane Ca^2+^ pump driven by Na^+^ influx rather than by ATP, the Na^+^/Ca^2+^ exchanger, but in many nerve cells the PMCA is the principle Ca^2+^ extrusion mechanism at low Ca^2+^ loads [4,7,9,10].

In snail neurones the PMCA is the only mechanism for calcium extrusion from the cell body [11], even though it is sometimes described as a low-capacity system [3]. The ratio of the coupling between Ca^2+^ extrusion and H^+^ uptake at normal pH levels by the PMCA remains controversial. While the early workers [1] concluded that the Ca^2+^:H^+^ ratio was 1:2, some subsequent work has suggested fewer H^+^ ions are transported [12–14]. A recent review [2] states categorically that the ratio is 1:1, as does a recent physiology textbook [15]. Many biochemistry textbooks still report that the PMCA is a uniporter (see [14]). There have also been several reports that the PMCA is electrogenic, for example in hair cells [16] and in red blood cell preparations [17,18]. Electrogenicity implies that the coupling is not 1Ca^2+^:2H^+^. In contrast, in snail neurones I recently found that the ratio under normal conditions is 1Ca^2+^:2H^+^ [19].

The effects of extracellular pH (pH_o_) on the coupling ratio have been studied in red blood cells and barnacle muscle, but not in nerve cells. In red blood cell PMCA preparations the Ca^2+^:H^+^ ratio is changed by pH_o_ from about 1:2 at pH 6.5 to 1:0 at pH 8.5 [20]. Similarly in barnacle muscle at external pH 6.5 the PMCA coupling ratio was 1:3, while at pH 8.2 the ratio was 1:1 [21]. The effects of changes in intracellular pH (pH_i_) have not apparently been investigated in any cell. The quantity of H^+^ ions pumped into neurones by the PMCA is important because many channels and carriers are sensitive to small pH changes [22,23]. The possible pH sensitivity of the coupling ratio may also have important consequences for the molecular mechanism of the PMCA.

To investigate the effects of pH on the coupling ratio in nerve cells I have extended my recent experiments [19] on the large neurones of the common snail *Helix aspersa.* Using both conventional and ion-sensitive microelectrodes I have recorded membrane potential, clamp currents, intracellular and surface pH and intracellular Ca^2+^ in intact cells. The PMCA was stimulated by depolarisation in Ca^2+^ or Ba^2+^ solutions or by direct iontophoretic injection of Ca^2+^. The subsequent PMCA-generated surface pH changes (ΔpH_s_) were measured. The two other processes that might change surface pH, namely intracellular pH regulation and H^+^ channels, were inhibited or kept inactivated. Normal pH_i_ regulation was inhibited by removal of bicarbonate [24] which leaves only a relatively weak Na^+^/H^+^ exchanger [25]. H^+^ channels were kept closed by avoiding large depolarisations [26]. To ensure that injected Ca^2+^ was not taken up by mitochondria, cells were injected with ruthenium red [27] or Ru360 [28]. This proved unnecessary for cells loaded by depolarisation. For the experiments on the effects of pH_o_ I measured the ΔpH_s_ for equal depolarisations or Ca^2+^ injections at different pH_o_ values. For the experiments on pH_i_ effects I measured the ΔpH_s_ induced by equal Ca^2+^ injections while changing pH_i_ by HCl injection or application and removal of CO_2_/bicarbonate. With pH_o_ between 7.5 and 8.5 I have found significant changes in the PMCA-induced ΔpH_s_ only with injected Ca^2+^ loads. Allowing for variation in the pump rate with pH, these findings suggest that the snail neurone PMCA coupling ratio is little changed by external or internal pH.

## 2 Methods

### 2.1 General

Experiments were done on large (150–250 μm diameter) neurones in isolated sub-oesophageal ganglia of the common snail, *H. aspersa* [8,19,29]. Cells were voltage-clamped to −50 mV using two microelectrodes. The PMCA was stimulated either by depolarisations in Ca^2+^ or Ba^2+^ snail Ringer's solution, or by iontophoretic injection of Ca^2+^ ions. The changes in surface pH (ΔpH_s_) resulting from the subsequent extrusion of Ba^2+^ or Ca^2+^ were measured using pH-sensitive microelectrodes which were pressed against the surface of the chosen cell. The effects of changing pH_o_ were assessed by changing the superfusate pH using a mixture of three different buffers (with different dissociation constants) to minimise changes in buffering power. To reduce pH_i_, H^+^ ions were injected by iontophoresis, while to increase pH_i_ the preparation was first equilibrated with bicarbonate-buffered saline and then returned to normal snail Ringer's solution. The consequent loss of accumulated intracellular bicarbonate as CO_2_ caused a pH_i_ increase of 0.4–0.5 units. In some experiments changes in intracellular Ca^2+^ were followed with a Ca^2+^-sensitive microelectrode (CaSM).

### 2.2 Preparation

An aestivating snail was killed by rapid removal of the circumoesophageal ring of ganglia, and the large cells on the dorsal side of the suboesophageal ganglia exposed as previously described [19]. All experiments were carried out at room temperature, 18–23 °C, starting at least 1 h after the dissection.

### 2.3 Solutions

The normal snail Ringer solution contained (mM): 80 NaCl, 4 KCl, 7 CaCl_2_, 5 MgCl_2_, and 20 Hepes, titrated with NaOH to pH 7.5. Solutions of pH 6.5, 7.5 and 8.5 with a low buffering power had 2 or 5 mM of Pipes, Hepes and Taps, with additional NaCl to maintain tonicity. pH 9.5 Ringer was buffered with 20 mM CHES. Ba Ringer solutions had the same ionic composition but with BaCl_2_ replacing CaCl_2_. The CO_2_ Ringer solution was the same as normal except that it had 20 mM NaHCO_3_ instead of Hepes, was bubbled with 2.5% CO_2_ in air and contained 0.1 mM NaH_2_PO_4_.

Ruthenium red was dissolved in 0.1 M KCl at 10 mg ml^−1^ [31], and Ru360 was made up at 500 nM in 0.1 M KCl with 0.1% Fast green FCF to make it visible when injected. Aliquots of 0.1 ml of the solution were kept frozen until use. Ruthenium red injection pressure-injection electrodes often blocked, so Ru360 was preferred in later experiments. Both compounds had the same effects.

### 2.4 Microelectrodes

Conventional micropipettes were pulled from 1.2 mm filamented borosilicate glass tubing and backfilled with 1 M CsCl for passing clamp current or recording membrane potential. Microelectrodes for iontophoretic injection were filled with 0.1 M CaCl_2_ with tips broken by touching a pin in the bath to give resistances of 5–10 MΩ. For H^+^ injection microelectrodes were pulled from quartz tubing filled with 1 M HCl and left with tips intact.

Intracellular CaSMs were made from quartz glass and pHSMs from borosilicate glass [30,31]. For surface pH, both liquid ion sensor and Hinke-style glass pH microelectrodes [32] were used. The latter had the advantage of recording from a large area, and being less likely to penetrate the cell membrane.

### 2.5 Data collection and analysis

Potentials from the conventional microelectrodes, CaSMs and pHSMs were recorded as voltages referred to membrane potential, with the voltages from the pHSMs converted to pH before display. Potentials from the surface pHSMs were referred to the bath potential. Results were discarded if on withdrawal of an electrode its potential in Ringer solution had changed by more than 7 mV.

Potentials from the voltage-recording microelectrodes were led via preamplifiers in the Faraday cage to an 8-pole Bessel filter and recorded at 20 Hz on a PC via a CED micro 1401 interface and Spike 2 data collection program (Cambridge Electronic Design, UK). The clamp current was recorded at 100 Hz as above.

Figures were prepared from the CED data after loading into Microsoft Excel. Spikes in the *V*_Ca_ or pH records generated by electronic pickup were partially erased, and the clamp current records were in some cases restricted in range. Data are presented as means ± S.E.M. of *n* observations. The statistical significance of observed differences was determined by a paired two-sample two-tailed *t*-test for means. Differences between means were considered significant when *P* < 0.05.

## 3 Results

### 3.1 Ca^2+^ uptake by mitochondria or extrusion by the PMCA

The PMCA is continually active, maintaining the normal low [Ca^2+^]_i_ in the face of a continuous influx even in a cell voltage-clamped at −50 mV. To investigate the coupling between Ca^2+^ extrusion and H^+^ uptake I have chosen to stimulate the PMCA with a brief, repeated, additional load of Ca^2+^ or Ba^2+^ ions. I assume that the coupling does not change with rate of activity. A Ca^2+^ load can be delivered in several ways. The simplest way is by a brief depolarisation to open Ca^2+^ channels, while a second more controllable method is by iontophoretic injection of Ca^2+^ ions [32].

**Fig. 1:**
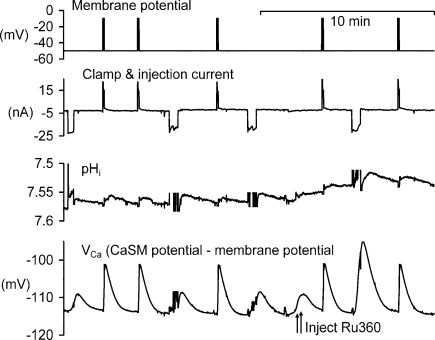
The effect of Ru360 injection on the responses to depolarisation and calcium injection. The transient changes in intracellular pH (pH_i_) and intracellular Ca^2+^ were generated by either depolarisations or iontophoretic injections of Ca^2+^. Ru360 was pressure-injected where indicated by the arrows. The top record shows membrane potential, clamped at −50 mV or −10 mV. The second record shows the current passed by the voltage clamp electrode to depolarise the cell (+ve current) or inject Ca^2+^ iontophoretically (−ve current). The third record shows intracellular pH recorded with a microelectrode. The bottom record shows intracellular Ca^2+^ as the potential (*V*_Ca_) recorded by an intracellular Ca^2+^-sensitive microelectrode. Electrical noise from the injection current seen on the pH and *V*_Ca_ recordings has been partially erased.

In initial experiments I measured the intracellular pH change resulting from the uptake of H^+^ while an injected Ca^2+^ load was extruded. With a number of assumptions, including that all the injected Ca^2+^ was extruded, I was able to assess possible changes in coupling ratio. This last assumption, however, proved untenable, as was indeed suggested by earlier work with pressure-injected Ca^2+^ [33]. My later experiments confirmed that some of the injected Ca^2+^, but not Ca^2+^ entering through channels, was taken up by mitochondria, as shown in Fig. 1. In this experiment I recorded both pH_i_ and *V*_Ca_ (the potential from the CaSM referred to the membrane potential) and stimulated the PMCA by both depolarisations and Ca^2+^ injections. The first three depolarisations each caused an increase in *V*_Ca_ of 13 mV (i.e. an increase in intracellular Ca^2+^) erased and a small fall in pH_i_. Similarly the two iontophoretic injections of Ca^2+^ each caused an increase in *V*_Ca_ of 5 mV. Although the pH_i_ decrease was larger for the two injections than for the depolarisations, the increase in *V*_Ca_ for the injections was smaller than for the depolarisations. I then injected Ru360, which blocks Ca^2+^ uptake by mitochondria [28]. This greatly increased the *V*_Ca_ increase seen with an injection, to 19 mV, but did not alter the depolarisation-induced Ca^2+^ increases. The pH_i_ responses were not obviously changed. (The injection of Ru360 itself had a small effect on pH_i_, and a similar but slower effect on *V*_Ca_, to those seen with the Ca^2+^ injections.) Similar results were obtained with ruthenium red. This suggests that a large fraction of the injected Ca^2+^ was taken up by the mitochondria.

**Fig. 2:**
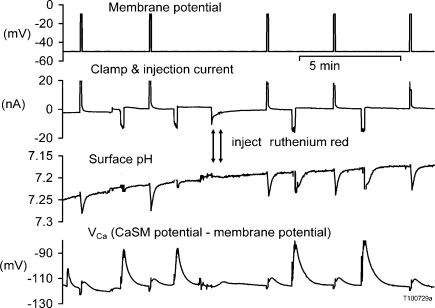
Ruthenium red magnifies surface pH responses to injections but not to depolarisations. Both surface pH and *V*_Ca_ were recorded while Ca^2+^ loads were applied in two ways. After two 40 mV depolarisations and two injections of Ca^2+^, ruthenium red was injected to make the cell visibly pink. Then three more depolarisations and two more injections were made. The baseline surface pH fell slowly during the experiment as the superfusate pH was very weakly buffered.

Mitochondrial Ca^2+^ uptake blockers also increased the surface pH changes induced by injection. Such ΔpH_s_ result from the H^+^ being pumped into the cell as the Ca^2+^ is extruded [5,19]. The size of the ΔpH_s_ depends on the location of the pH electrode, the local buffering power and the activity of the PMCA. If most of the injected Ca^2+^ is taken up by the mitochondria there will be little surface pH increase, as shown in Fig. 2. In this experiment the cell was alternately depolarised and injected with Ca^2+^, and both pH_s_ and *V*_Ca_ measured. About 7 min after the start of the recording shown, ruthenium red was injected sufficient to make the cell clearly pink. (From the size of the bolus of ruthenium red seen at the end of each pressure-injection pulse I estimate that the final concentration was between 0.01 and 0.1 mM.) I then resumed alternating the methods of delivering a Ca^2+^ load. The Ru360 had little effect on either the ΔpH_s_ or Ca^2+^ increases following depolarisations, but increased both the Ca^2+^ transients and, spectacularly, the ΔpH_s_ following Ca^2+^ injections.

In a total of five similar experiments I found that blocking mitochondrial uptake increased the size of the *V*_Ca_ transient following injection by an average factor of 2.56 ± 0.57. In three experiments blocking uptake increased the surface pH changes by an average of 2.3 ± 1.1 times. On the other hand the blockers had no effect on the responses of *V*_Ca_ and surface pH to depolarisations. Presumably injections deliver a high concentration of Ca^2+^ deep in the cell, close to the mitochondria, while entry through channels delivers a lower concentration of Ca^2+^ all round the cell periphery, where the PMCA is located. I have therefore subsequently done experiments with Ca^2+^ injections only on cells injected with ruthenium red or Ru360.

I have also tested cyclopiazonic acid (CPA), which blocks Ca^2+^ uptake by the endoplasmic reticulum. It was without effect, after stores were empty, on any of the responses to depolarisation or injection (see also [8]). It therefore seems likely that under the conditions of my experiments, the endoplasmic reticulum plays no significant role in the activity of the PMCA. I have assumed that other intracellular organelles play no significant role in sequestering Ca^2+^. In several experiments I have tested the effects of Ru360 or ruthenium red on internal Ba^2+^ responses to iontophoretic injection. The responses were unchanged, suggesting that Ba^2+^ is not taken up by snail neurone mitochondria (data not shown).

### 3.2 The effect of external pH on surface pH changes caused by activation of the PMCA by Ca^2+^ or Ba^2+^ influx

**Fig. 3:**
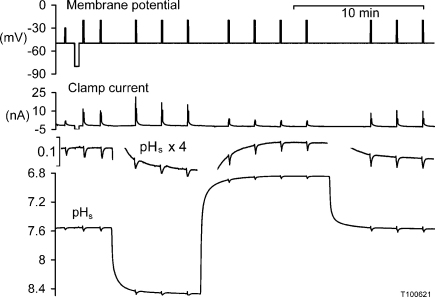
The effect of increasing or decreasing external pH on the surface pH responses to Ca^2+^ entry. After the first 2 min in normal Ringer and a test of the ion-sensitive microelectrodes’ insensitivity to a brief hyperpolarisation, the preparation was superfused with a Ringer of the same composition but buffered to three different pH values, with 5 mM of Pipes, Hepes and TAPS. (These three buffers provide a nominally constant buffering power over the pH range 6.5–8.5.) The surface pH changes are also shown enlarged as inserts above the continuous recording of surface pH.

Fig. 3 shows part of an experiment in which I applied a series of 5 s, 30 mV depolarisations while recording the surface pH. The nominal pH of the low-buffered superfusing Ca^2+^ solutions was 7.5, 8.5 and 6.5. Each depolarisation initiated a ΔpH_s_ of about 0.025 units. Both the increase and decrease in superfusate pH from normal pH 7.5 appeared to reduce the size of the surface pH changes by 5–15%.

**Fig. 4:**
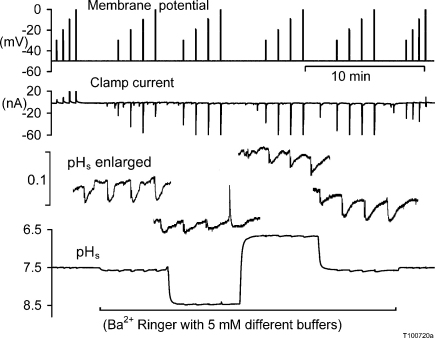
Surface pH changes following Ba^2+^ influx at different external pH. The cell was depolarised for 5 s by 20, 30, 40 and 50 mV in different solutions. The first solution was normal Ca^2+^ snail Ringer with 20 mM Hepes at pH 7.5. After 4 min this was changed to a series of Ca^2+^-free Ba^2+^ Ringers buffered to three different pH values with 5 mM Pipes, Hepes and TAPS. Sections of the enlarged pH_s_ trace on a faster time base are shown above the continuous record.

Fig. 4 shows part of a similar experiment done in Ba^2+^ Ringer after an initial period in normal Ca^2+^ Ringer. Ba^2+^ is extruded by the PMCA more slowly than Ca^2+^ [19], so the surface pH changes were sometimes easier to measure. Since Ba^2+^ does not activate K^+^ channels, I was also able to estimate the Ba^2+^ influx from the charge needed to depolarise the membrane. In this experiment the depolarisations were of 20, 30, 40 and 50 mV for 5 s in each series, with the first and last series in normal Ca^2+^ Ringer. The increasing size of the depolarisations was designed to reveal at what potential the H^+^ channels opened. Again the ΔpH_s_ were smaller in pH 6.5 and 8.5 than in 7.5, but the transient caused by the largest depolarisation in pH 8.5 were contaminated with H^+^ efflux through channels, as shown by the brief surface acidification.

**Fig. 5:**
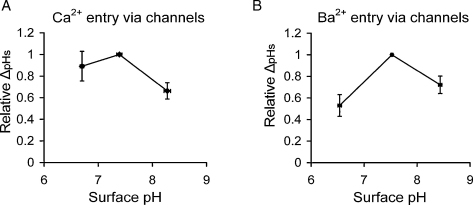
Collected results for the surface pH effects of Ca^2+^ (six experiments, Fig. 5A) or Ba^2+^ (five experiments, Fig. 5B) entry at different surface pH values. The peak change in surface pH induced by depolarisation for 5 s by 30 or 40 mV at each value of external pH was averaged for each experiment, and these averages were expressed relative to those at pH 7.5, and plotted against the average measured surface pH. The error bars are S.E.M.s. None of the values are significantly different.

The results from six experiments in low buffered Ca^2+^ and five in low buffered Ba^2+^ Ringer are compared in Fig. 5. In each case I have measured the peak size of the ΔpH_s_ responses to the same size depolarisation (usually 30 mV), averaging several measurements at each pH where possible. I have then calculated the ΔpH_s_ for pH 6.5 and 8.5 as ratios of that seen at pH 7.5, and averaged these ratios for all experiments. The results show that both a pH increase and a decrease from normal apparently reduce the size of the surface pH changes, although not significantly.

One factor that may contribute to this is a reduced Ca^2+^ or Ba^2+^ entry at pH 6.5, since Ca^2+^ channels are well-known to be inhibited by low pH [34]. Unfortunately it is not possible to determine the size of the Ca^2+^ influx from the clamp current, since Ca^2+^ entry activates K^+^ channels. With Ba^2+^ entry, however, few K^+^ channels are opened, so the charge carried in by the Ba^2+^ is likely to be close to the total clamp current during the depolarisations [19]. I have therefore measured the charge carried in the experiments averaged in Fig. 5B. At pH 6.5, 7.5 and 8.5 the average charges were 180 ± 27; 218 ± 32 and 230 ± 33 nC, respectively. This suggests that at pH 6.5 the Ca^2+^ influx is likely to be about 20% less than at pH 7.5.

### 3.3 The effect of external pH on surface pH and *V*_Ca_ changes caused by Ca^2+^ injection

**Fig. 6:**
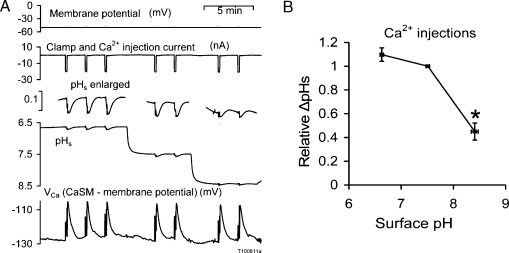
The effect of external pH on the changes in surface pH and *V*_Ca_ following Ca^2+^ injections. *A* shows part of an experiment, while *B* collects the results from a total of 6 similar experiments. The means for pH 8.4 are significantly different from those for pH 6.5 and 7.5.

The quantity of Ca^2+^ injected by a given charge is unlikely to be influenced by external pH, so any variation in ΔpH_s_ with pH will probably reflect a change in PMCA rate of activity or coupling between Ca^2+^ extrusion and H^+^ uptake. Fig. 6A shows part of an experiment in which I recorded both pH_s_ and *V*_Ca_ responses to a series of Ca^2+^ injections made at different external pH values. The cell had been earlier injected with ruthenium red. The peak ΔpH_s_ occurred some seconds after the end of the injection, since the injection site was deep in the cell and the diffusion of ions from the injection site is likely to take some time [29]. The pH_s_ responses at pH 8.5 were clearly smaller than at pH 7.5, and were followed by an acid overshoot. Fig. 6B collects the results from a total of 6 similar experiments. The responses at pH 6.5 and 7.5 were similar, but those at pH 8.5 were much smaller than at pH 7.5.

**Fig. 7:**
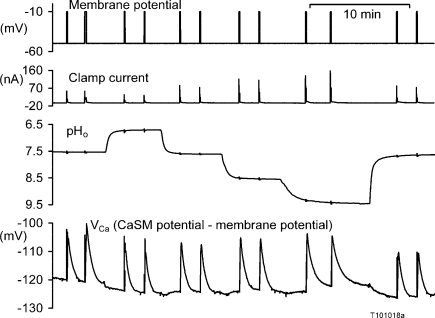
The effect of external pH on Ca^2+^ recovery after depolarisations. The effect of different external pH solutions superfused over a snail neurone on the response of intracellular Ca^2+^ to repeated depolarisations to −10 mV from the holding potential of −50 mV. All depolarisations were for 5 s, except for the second which was for 10 s.

**Fig. 8:**
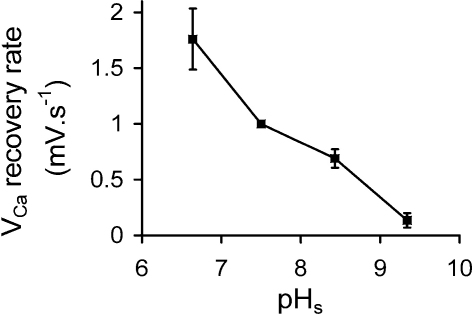
The effect of external pH on the *V*_Ca_ recovery rate. The rate of recovery of intracellular Ca^2+^ (as *V*_Ca_) at 4 different external pH values, from 6 experiments. Means ± S.E.M. For each experiment a value of *V*_Ca_ was selected which was included in all the transients, and the slope of the *V*_Ca_ record at the potential measured. Values for each experiment were related to the recovery rate at pH 7.5.

Part of the effect of high pH described so far probably results from inhibition of the PMCA by the reduction in available H^+^ [4,5]. I have therefore measured the effects of a wide range of external pH values on the rate of recovery of *V*_Ca_ after depolarisation-induced Ca^2+^ loads. Fig. 7 shows an example. I analysed the results from this and five other similar experiments by measuring the slope of the *V*_Ca_ record at a value of *V*_Ca_ which passed through all the recoveries (*V*_Ca_ = −115 for the experiment of Fig. 7). I have plotted the rates of recovery relative to those seen at pH 7.5 in Fig. 8. It is clear that a high external pH inhibits the pump, with the rate at pH 8.5 being on average 69% of that at pH 7.5.

### 3.4 The effect of changes in intracellular pH on pHs changes following Ca^2+^ injection

**Fig. 9:**
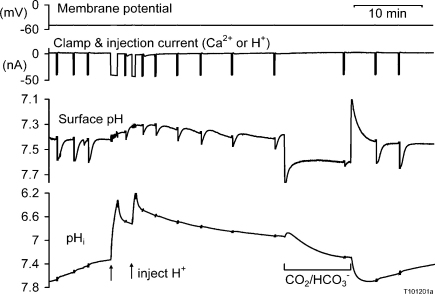
The effect of changing pHi on the surface pH response to a series of Ca^2+^ injections. The Ca^2+^ injections were all made by a current of 40 nA passed for 5 s, while pHi was changed by either injecting HCl or by superfusion with a 2.5% CO_2_/HCO_3_^−^ buffered solution. Otherwise the preparation was superfused with a solution buffered to pH 7.5 with 5 mM Hepes, Pipes and TAPS.

The effects of pH_i_ on the PMCA coupling ratio do not seem to have been investigated before. To test the effects of pH_i_ on the PMCA H^+^ uptake I either reduced pH_i_ by injecting H^+^ ions iontophoretically or increased pH_i_ by a period of superfusion with CO_2_/bicarbonate buffered Ringer's solution. A representative experiment is illustrated in Fig. 9. After three Ca^2+^ injections at pH_i_ between 7.7 and 7.4, two injections of H^+^ lowered pH_i_ to about 6.6. At this low pH_i_ Ca^2+^ injections caused greatly reduced ΔpH_s_ which were followed by a large pH_i_ overshoot beyond the pre-injection level. As pH_i_ increased, so did the responses to Ca^2+^ injection. *V*_Ca_ was not recorded in this experiment, but in others and as reported earlier [8], low pH_i_ caused an increase in the baseline *V*_Ca_.

**Fig. 10:**
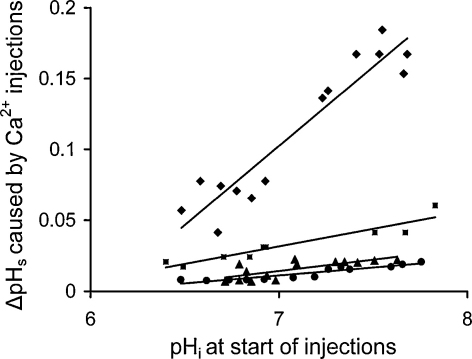
Intracellular acidification reduces the effect of Ca^2+^ injections on surface pH. Results from a total of 4 cells (plotted with different symbols) including that shown in Fig. 9. Data for each cell has been fitted by a least-squares line.

Fig. 10 gives the results from four experiments on the effects of different pH_i_ on the ΔpH_s_ caused by Ca^2+^ injections. All cells showed that low pH_i_ reduced the pH_s_ responses by a factor of about 2 over a range of 1 pH unit. I have previously shown that low pH_i_ slows the *V*_Ca_ recovery rate [8]. Allowing for this, the changes in surface pH with pHi were not significant.

## 4 Discussion

My results show that depolarisations provide a consistent way of stimulating the PMCA, which generates surface pH increases as H^+^ ions are pumped in. Similarly, Ca^2+^ injections are also effective so long as mitochondrial uptake is blocked. For depolarisation-induced surface pH changes I found no significant effect of altering external pH. The Ca^2+^ injection-induced surface pH transients were reduced by 55% by high external pH, although much of this can be accounted for by the average *V*_Ca_ recovery rate being 31% slower than at pH 7.5. These results suggest that the apparent effects of external or intracellular pH on the surface pH changes caused by activating the PMCA were likely to result from changes in the pump rate or measurement error rather than changes in the coupling ratio. Any effects on the coupling ratio were at most much smaller than previously reported. (Since it is not known how many different PMCAs might be active in snail neurones, my results strictly apply only to the total PMCA activity.)

The validity of my measurements, and their relevance to the PMCA coupling ratio, depends on a number of assumptions. These concern the constancy of the Ca^2+^ load and the extent of its extrusion by the PMCA, the accuracy of its measurement with a CaSM at a point in the cell, and the extent to which the surface pH change is proportional to the H^+^ uptake. I also assume that the PMCA coupling ratio does not change with rate of activity, since this has not been reported for any ATPase. I have made no attempt to measure the coupling ratio itself, being concerned only with its possible alteration.

### 4.1 Possible changes in the Ca^2+^ load and its extrusion

I have assumed that repeated depolarisation-induced loads are relatively unaffected by time or pH. From the measurements of the charge carried by Ba^2+^ it is likely that low pH_s_ reduces Ca^2+^ influx; the sensitivity of Ca^2+^ channels to pH has indeed been known for many years [34]. I have also assumed that iontophoretic injections of Ca^2+^ by the same charge are not changed by either pH or time. Following my experiments with ruthenium red and Ru360, I have assumed that the pH_s_ increase generated by a depolarisation-induced Ca^2+^ or Ba^2+^ load was generated only by the PMCA. Similarly, where a cell has been injected with Ru360 or ruthenium red I assume that most of the injected Ca^2+^ is rapidly extruded, and not sequestered. In previous work with very large pressure injections of CaCl_2_ it was concluded [33] that the pH_i_ decreases observed were generated largely by mitochondrial Ca^2+^/H^+^ exchange with some possible contribution from Ca^2+^ extrusion. Others have reported that mitochondria are involved in buffering Ca^2+^ loads in rat neurones [35].

Previous recordings of intracellular Ba^2+^ and Ca^2+^ recoveries after injection or loading by depolarisation [8,19] support the assumption that essentially all the unsequestered divalent cation load is pumped out by the PMCA, within 1–2 min of the end of the injection or depolarisation. This correlates with the time-course of the ΔpH_i_. Such an assumption cannot be relied on, however, at low pH_i_ values: my previous recordings of *V*_Ca_ show that at low pH_i_ values the PMCA does not always return the *V*_Ca_ to its pre-injection level.

I have shown that while Ca^2+^ uptake by the endoplasmic reticulum is little changed by intracellular acidification, release is inhibited [8]. This might change the proportion of injected Ca^2+^ that is rapidly pumped out by the PMCA, but I found no effect of CPA, which blocks Ca^2+^ uptake by the endoplasmic reticulum. All my measurements on the effects of pH_i_ were made as pH_i_ fell, so that release from the endoplasmic reticulum would be unlikely while I was measuring the injection-induced ΔpH_s_. I also found that CPA had no effect on the pH or *V*_Ca_ changes following Ca^2+^ loads.

### 4.2 Measurement of the Ca^2+^ extrusion rate

To assess the PMCA activity I measured the recovery of *V*_Ca_ after an injection or a depolarisation. The *V*_Ca_ was recorded at a single point inside each neurone, close to the cell membrane on the far side of the cell. Thus its recovery can be only an indirect measure of pump rate. Previous experiments with the CaSM method do suggest, however, that the decline in *V*_Ca_ can be fitted closely by models including intracellular diffusion and pump rate [29].

### 4.3 pH_s_ changes as a measure of H^+^ uptake

As well as the PMCA, two other membrane pathways may influence surface pH. The first, pH_i_ regulation, was active only at a low level since bicarbonate was absent during PMCA activation. (I added bicarbonate only to accelerate pH_i_ recovery from large acidifications, as in Fig. 9.) The pH_s_ overshoots seen at pH_o_ 8.5 in Fig. 6 may have been due to pH_i_ regulation activated by the transiently large H^+^ gradient before the H^+^ diffused away from the interior of the cell membrane. They suggest that residual pH_i_ regulating mechanisms may be driven by a local pH gradient caused by the PMCA. If this was activated rapidly it would reduce the size of the observed ΔpH_s_. I avoided using inhibitors of pH regulation since they are only slowly reversible and tend to have multiple effects. The second non-PMCA pathway is the H^+^ channel, opened by large depolarisations [26]. At pH_o_ 8.5 I did see evidence of H^+^ channel opening, e.g. in Fig. 4 with the 50 mV depolarisation. The H^+^ efflux produced a brief spike in pH_s_ which may well have reduced the total PMCA effect. Measurements showing such signs of H^+^ efflux were excluded from the analysis. From experiments on surface pH reported earlier [36] it is clear that pH_s_ changes produced by very large depolarisations are proportional to H^+^ flux across the cell membrane via H^+^ channels and to external buffering power. Previously such measurements were made at essentially the same pH. Those reported here, however, were done in three solutions of different pH using buffers with p*K* values of 6.8, 7.5 and 8.4. These gave a relatively constant buffering power between pH 6.5 and 8.5. Thus the same ΔpH_s_ in this range should represent that same flux of H^+^ ions. This makes no allowance for a possible contribution of fixed buffers on the cell surface, about which little is known. Perhaps they make a significant contribution at low pH values.

If the reduction in PMCA activity at high pH explains in part the reduction in the ΔpH_s_ at pH 8.5, then the increased pump rate at pH 6.5 should theoretically make the ΔpH_s_ much larger. Failure to demonstrate this possibly suggests that the peak rate of the PMCA, which determines the size of the ΔpH_s_, was limited by availability of ATP or another factor.

### 4.4 Comparison with previous measurements

These findings differ from those reported for the effects of extracellular pH on the coupling ratio of the PMCA of red blood cells and barnacle muscle fibres. With red blood cells it was reported that the number of H^+^ ions pumped in for each Ca^2+^ extruded was about 1.9 at pH_o_ 6.3, but that this number fell to zero as pH_o_ was increased to 8.0 [20]. In other words the more H^+^ that were available outside the cell, the more were pumped in. With barnacle muscle similar findings have been reported [21]. At a pH_o_ of 6.5 the authors estimated that 3H^+^ were taken up for each Ca^2+^ extruded, while at pH 8.2 only 1H^+^ ion was pumped in. No previous study has been published on the effects of pH_i_ on the coupling ratio.

The sensitivity of the PMCA to pH may well be different in neurones. It is also worth noting that my methods were quite different from those used previously, and perhaps involved less disruption to the normal physiology of the cell. Both previous reports measured Ca^2+^ efflux with ^45^Ca. For the red blood cell studies the H^+^ uptake of a large number of cells was measured by clamping extracellular pH and measuring how much acid must be added as the ^45^Ca was pumped out [20]. With barnacle muscle the pH_i_ of a single muscle fibre was followed with a microelectrode, and measured buffering power used to estimate H^+^ uptake [21]. A variety of treatments was used to block all pathways, except the PMCA, that might allow Ca^2+^ or H^+^ movement across the cell membrane. External Na^+^ and Ca^2+^ were both removed. My experiments were done with normal Na^+^ and Ca^2+^ gradients, on single voltage-clamped cells to minimise passive ion fluxes. Cells were unavoidably loaded with Cl^−^ via the microelectrodes, but it is hard to see how this would influence the PMCA.

If my assumptions are correct, I conclude that in my preparation the PMCA coupling ratio is relatively insensitive to changes in either intracellular or extracellular pH. This has important implications for the power of the PMCA as a source of intracellular H^+^, as well as for its molecular mechanism.

## Conflict of interest

There are no conflicts of interest.
